# Scarlet Fever

**Published:** 1860

**Authors:** J. W. Benson

**Affiliations:** Prof. of Anatomy in the University of Louisville


*Scarlet Fever.* By J. W. BENSON, M. D., Prof. of Anatomy in the University of Louisville.

In twenty-five successive cases of this disease, which have been latterly under my professional care, the treatment consisted in inunction of the parotid and submaxillary regions, by an unguent composed of fifteen grains of the extract of belladonna to an ounce of simple ointment. This was applied freely and frequently, as soon as the patient complained of sore throat. A piece of flannel was afterwards applied, and in no case was any other treatment adopted, except the administration of small quantities of neutral mixture during the day. In some cases of rapidly occurring tumefaction of the throat, the prompt subsidence thereof under the treatment, left no room for doubt as to its efficacy. I do not pretend to offer this mode of treatment either as a cure for Scarlet Fever, or as the sole means to be relied upon in any case, but I do claim for it a controlling power over the engorgement, and hence a prevention of those destructive ulcerations of the throat, which are so much and so justly dreaded. In some cases it has seemed to have a salutary effect upon existing diarrhœa, as soon as the system was influenced by the remedy.

In one case only was I compelled to discontinue its use because of its constitutional effect. I will not here discuss its modus operandi, but simply suggest that the experiments of physiologists in reference to the influence of the organic nerves upon glandular organs, coupled with an experience of thirteen years in its use as a restraining remedy in salivation, and a more limited but somewhat extensive observation of its influence on the mammary gland, seemed to justify, on purely rational and philosophical grounds, the adoption of the course pursued.

During a discussion, some months ago, in the College of Physicians and Surgeons, upon the merits of belladonna treatment in profuse lactation and mammary inflammation, I took the liberty of intimating, that perhaps the contradictory results of the observation of members might have obtained from a failure to distinguish between the pathological condition of the gland itself, and that of the areolar structure in relation with it, for if my views of its action be correct, it might not influence directly the latter condition, but would prove potent in the former. Since the results of the application, as indicated, were reported to the College, some of my friends have adopted the same course, and with the same results, viz., perfect success in every case.

They, therefore, concur with me in attributing such results to something else than mere coincidences or negative effects. They may not be, but the application is a simple, and, under judicious watchfulness, a harmless one, and I will be as free to confess its inertness, as I am now anxious to press its claims to attention, so soon as my duty shall seem to indicate such a course.—*Louisville Monthly Medical News.*

